# Genome Sequence of Vibrio cholerae Strain D1, Isolated from the Maumee River in Toledo, Ohio

**DOI:** 10.1128/MRA.01312-18

**Published:** 2018-10-25

**Authors:** Jyl S. Matson

**Affiliations:** a Department of Medical Microbiology and Immunology, University of Toledo, Toledo, Ohio, USA; University of Arizona

## Abstract

Vibrio cholerae is a human bacterial pathogen and an inhabitant of aquatic environments. It is endemic to many regions of the world but is typically found in warm climates in saltwater. Here, we present the sequence of a V. cholerae strain isolated from a freshwater river in Ohio.

## Abstract (precis)

Vibrio cholerae is a human bacterial pathogen and an inhabitant of aquatic environments. It is endemic to many regions of the world but is typically found in warm climates in saltwater.

## ANNOUNCEMENT

*V*ibrio cholerae is the bacterium responsible for the epidemic diarrheal disease cholera, but it is also a natural inhabitant of aquatic environments in many regions of the world (1). V. cholerae is typically found in estuarine or coastal environments, with most strains residing in subtropical climates, such as Southeast Asia. In the United States, V. cholerae can be isolated from coastal environments, such as the Chesapeake Bay (2). Typically, these strains are nontoxigenic (do not produce the cholera toxin) (3), but several studies have shown that there is a high degree of genetic diversity in the isolates and they contain a random assortment of virulence genes that may warrant increased surveillance (4).

Recently, I have found that there are environmental V. cholerae strains present in freshwater lakes and rivers near Toledo, Ohio (unpublished data). As most vibrios prefer at least a small amount of salt in their habitat and are typically found in warmer climates, this is unexpected. In order to learn more about these North American strains of V. cholerae, we sequenced one of its isolates. V. cholerae isolate D1 was obtained from a surface water sample taken from the Maumee River on 29 September 2017. The collection location is 41°33′37″N, 83°37′53″W. In order to enrich for Vibrio strains, the water was filtered through a 0.2-µm membrane, and the filter paper was then incubated in alkaline peptone water (APW), pH 8.6 (5). After incubation, aliquots from the surface of the medium were plated on thiosulfate-citrate-bile salts-sucrose (TCBS) agar, a selective medium for Vibrio isolates. Yellow colonies that grew on TCBS were isolated and subsequently screened by PCR for the presence of the V. cholerae *ompW* gene, a species-specific diagnostic marker for V. cholerae (6).

To prepare for sequencing, a single colony of V. cholerae isolate D1 was grown overnight at 37°C on LB agar, and genomic DNA was isolated using the Qiagen QIAamp DNA minikit (Valencia, CA). Genome sequencing of the strain was performed by SNPsaurus (Eugene, OR) using PacBio technology (Menlo Park, CA). Sequencing coverage was 147.01×, and the average read length was 10,052 bp. Sequences were assembled into three contigs using Canu v.1.7 (default parameters) (7), for a total of 4,139,713 bp. The three contigs are 2,972,158, 1,159,947, and 7,690 bases in size. The 1,159,947-bp contig was predicted to be circular by Canu. The first two contigs likely represent the large and small V. cholerae chromosomes, respectively, based on their size (8). The first contig is most similar to V. cholerae strain MS6, and the second is most similar to strain 10432-62. No sequences corresponding to the *ctxAB* genes were identified, suggesting that this is a nontoxigenic isolate.

### Data availability.

This whole-genome shotgun project has been deposited at DDBJ/ENA/GenBank under accession number QZCU00000000. The version described in this paper is version QZCU01000000. Raw sequences were deposited in the NCBI SRA database under BioProject number PRJNA491705.
